# Comparative evaluation of displacement and stress distribution pattern during mandibular arch distalization with extra and inter-radicular mini-implants: a three-dimensional finite element study

**DOI:** 10.1590/2177-6709.28.2.e2321373.oar

**Published:** 2023-05-29

**Authors:** Amit MAHESHWARI, Dhruv Nilesh CHAWDA, Ashish KUSHWAH, Rajesh Kumar AGARWAL, Amesh Kr GOLWARA, Prateek Bhushan DIXIT

**Affiliations:** 1 JMF’s ACPM Dental College, Department of Orthodontics and Dentofacial Orthopedics (Dhule/Maharastra, India).; 2 People’s College of Dental Sciences and Research Centre, Department of Orthodontics and Dentofacial Orthopedics (Bhopal/Madhya Pradesh, India).; 3 Private practice (Kokomo, USA).; 4 Buddha Institute of Dental Sciences and Hospital, Department of Orthodontics and Dentofacial Orthopedics (Patna/Bihar, India).; 5 Teerthanker Mahaveer Dental College, Department of Orthodontics and Dentofacial Orthopedics, Post-graduate program (Moradabad/Uttar Pradesh, India).

**Keywords:** Arch distalization, Buccal shelf implant, Extra-radicular, Inter-radicular, Finite element method

## Abstract

**Objective:**

To compare the initial stress distribution and displacement on mandibular dentition using extra and inter-radicular mini-implants for arch distalization, by means of finite element analysis.

**Methods:**

For this study, two finite element models of the mandible were designed. The models consisted of periodontal ligament (PDL) and alveolar bone of all teeth until second molars. In the Case 1, bilateral extra-radicular buccal-shelf stainless steel mini-implants (10.0-mm length; 2.0-mm diameter) were placed between first and second permanent molars. In the Case 2, bilateral inter-radicular stainless steel mini-implants (10.0-mm length; 1.5-mm diameter) were placed between second premolar and first permanent molar. Power hook was attached between canine and first premolar at a fixed height of 8mm. In the two cases, 200g of distalization force was applied. ANSYS v. 12.1 software was used to analyze and compare von Mises stress and displacement in the mandibular dentition, PDL and bone.

**Results:**

Higher stresses were observed in mandibular dentition with the inter-radicular implant system. The amount of von Mises stress was higher for cortical bone (85.66MPa) and cancellous bone (3.64MPa) in Case 2, in comparison to cortical bone (41.93MPa) and cancellous bone (3.43MPa) in Case 1. The amount of arch distalization was higher for mandible in Case 1 (0.028mm), in comparison to Case 2 (0.026mm).

**Conclusion:**

Both systems were clinically safe, but extra-radicular implants showed more effective and controlled distalization pattern, in comparison to inter-radicular implants, in Class III malocclusion treatment.

## RESUMO

**Objetivo:**

Comparar a distribuição da tensão inicial e o deslocamento na dentição inferior usando mini-implantes extra e inter-radiculares para distalização da arcada, por meio da análise de elementos finitos.

**Métodos:**

Dois modelos de elementos finitos da mandíbula foram criados, os quais consistiram de ligamento periodontal (PDL) e osso alveolar de todos os dentes até os segundos molares. No Caso 1, mini-implantes extra-radiculares de aço inoxidável (10,0 mm de comprimento; 2,0 mm de diâmetro) foram colocados bilateralmente na *buccal-shelf* entre o primeiro e o segundo molares permanentes. No Caso 2, mini-implantes de aço inoxidável inter-radiculares (comprimento de 10,0 mm; diâmetro de 1,5 mm) foram colocados bilateralmente entre o segundo pré-molar e o primeiro molar permanentes. Um *Power hook* foi preso entre o canino e o primeiro pré-molar a uma altura fixa de 8mm. Nos dois casos, foi aplicada força de distalização de 200g. O *software* ANSYS v. 12.1 foi usado para analisar e comparar a tensão de von Mises e o deslocamento na dentição inferior, ligamento periodontal e osso.

**Resultados:**

Maiores tensões foram observadas na dentição inferior com o sistema de implantes inter-radiculares. A quantidade de tensões de von Mises foi maior para osso cortical (85,66MPa) e osso esponjoso (3,64MPa) no Caso 2, em comparação com osso cortical (41,93MPa) e osso esponjoso (3,43MPa) no Caso 1. A quantidade de distalização da arcada inferior foi maior no Caso 1 (0,028 mm), em comparação com o Caso 2 (0,026 mm).

**Conclusão:**

Ambos os sistemas foram clinicamente seguros, mas os implantes extra-radiculares mostraram um padrão de distalização mais eficaz e controlado, em comparação com os implantes inter-radiculares, para tratamento da má oclusão de Classe III.

## INTRODUCTION

Skeletal Class III malocclusion has relatively low incidence, with a prevalence of 14% in Asians, and 1-5% in Caucasians. The prevalence of Angle Class III malocclusion varies greatly among and within populations, ranging from 0% to 26%.^1^ It was found that the most common group of Class III patients comprises normal maxilla and overdeveloped mandible. However, a smaller group of patients is also seen with underdeveloped maxilla and overdeveloped mandible.^2^

As suggested by many authors, to correct anterior crossbite, mandibular anterior crowding, and mandibular dental asymmetry without extracting premolars, distalization of the mandibular teeth is the best treatment option.^3^ ^,^ ^4^ The options for this approach include using reverse headgears, chin cups, functional appliances and simple fixed appliance with heavy inter-arch elastics. The majority of the patients with severe skeletal Class III malocclusion are candidates for orthognathic surgery, which is the only choice to achieve a normal occlusion and an aesthetic profile. However, patient may not accept the surgery, and will continue to search for fixed orthodontic treatment.^5^

Class III elastics can cause unwanted side effects, such as maxillary incisor proclination, maxillary molar and mandibular incisor elongation, with tendency to expand maxillary molars, besides requiring patient compliance. To prevent these undesirable effects, absolute anchorage systems have been applied for either *en-masse* distalization of mandibular dentition or molar distalization.^6^

When using conventional intraoral distalizing appliances, there is an adverse and unavoidable reciprocal mesial movement of the anterior teeth and premolars during distal movement of the molars. Therefore, the resultant of the distalization process for the anterior segment is a round-trip movement.^7,8^ Distal movement using mini-implants allows the group movement of buccal segment teeth only: There is no forward movement of the anterior teeth in mini-implant supported biomechanics.^7^ ^,^ ^9^ ^,^ ^10^

For distalization of the mandibular dentition, mini-implants can be placed in various extra-radicular and inter-radicular sites. Elastomeric chain or NiTi close coil springs are attached to the mini-implants and the entire mandibular arch can be distalized or uprighted with minimal adverse effects.^1^

Inter-radicular mini-implants for distalization of the mandibular arch can be placed in various locations, such as between the mandibular second premolar and first molar or between the mandibular first molar and second molar.^7^ ^,^ ^11^ Also, extra-radicular implants like buccal shelf implants, lingual implants and retromolar pad implants, can be used for mandibular arch distalization.

The finite element method (FEM) has been utilized in Dentistry and Orthodontics due to its ability to evaluate stresses of interest, using computer-aided design (CAD) models. Finite element analysis (FEA) has particularly useful applications in evaluating aspects of mini-implants used in orthodontics.^12^ ^,^ ^13^

Both two-dimensional (2D) and three-dimensional (3D) stress analysis have been used to assess the dental implants. Many studies have made a comparison between the 3D and 2D FEA. The 3D method has been shown to offer a more precise prediction of stress distribution than the 2D method. Hence, distalization treatment effect can be compared with other models using different biomechanical variables, such as displacements, strains, and stresses, by means of the finite element models.^14^

No previous FEM study has evaluated the stress distribution on the mandibular teeth during distalization of the whole arch. Thus, the aim of this study was to analyze stress distribution pattern on the mandibular teeth during distalization with extra-radicular and inter-radicular mini-implants, using a 3D finite element model of the mandible.

## MATERIAL AND METHODS

Finite element analysis is a method for numerical analysis based on material properties. Finite element modeling is the representation of geometry in terms of a finite number of elements and their connection points, known as nodes, by building blocks for numerical representation of the model. The elements are of finite number, as opposed to a theoretical model with complete continuity. The object of interest has to be broken up into a meshwork that consists of a number of nodes on and in the object. These nodes, or points, are then connected to form a system of elements. By knowing the mechanical properties of the object, such as modulus of elasticity and Poison’s ratio, one can determine how much distortion each part of the cube undergoes when other part is moved by a force.^14^ ^-^ ^16^

**Figure 1:**
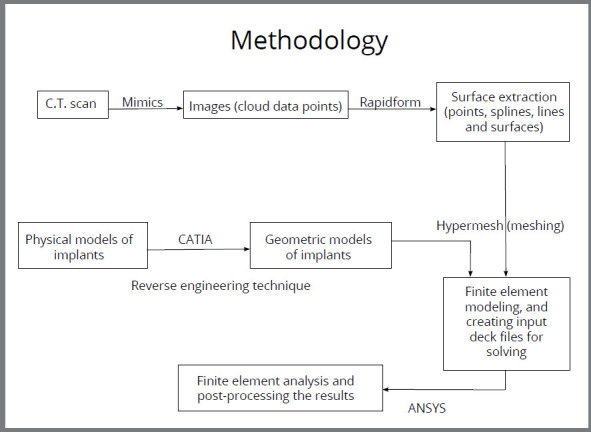
Flowchart of finite element analysis.

The methodology used for FEM analysis is described below and presented in Figure 1:

1. CT scan data of the mandible was taken as input, and was processed in MIMICS v. 8.11 software (Materialise‘s Interactive Medical Image Control System).
2. The required portion of mandible was considered and converted to STL format.
3. STL file was then converted to IGES data, using Rapid Form.
4. The geometric model in IGES format was further converted into finite element model using Hypermesh v.13.0 software.
5. In Hypermesh, separate modeling of bone, teeth, periodontal ligament and the implant-supported fixed appliance was done, and finally assembled together.
6. Material properties like elastic modulus and Poison’s ratio were assigned for teeth, periodontal ligament, bone, orthodontic brackets, implants and archwire.
7. Loads and boundary conditions were applied and finally linear static structural analysis was carried out using ANSYS v. 12.1 software. This system software is used for carrying out finite element analysis of structures and fluids for different purposes, as in automotive, civil, manufacturing, aerospace and biomedical fields, etc.
8. Results like displacement and stresses were processed for each material.

In this study, an analytical model of the mandible was developed. The model consisted of periodontal ligament and alveolar bone of all the teeth until second molars. Periodontal ligament with a mean thickness of 0.25mm was simulated around all teeth models. Mini-implants^17^ (1.5-mm diameter and 10-mm length, and 2-mm diameter and 10-mm length) and appliances metal parts, like brackets and archwire, were modeled using reverse engineering technique.

A MBT 0.022×0.028-in standard Edgewise metal brackets system^3^ was simulated and connected to the crowns so that the FA point was equal to the center of the bracket slot. Brackets were bonded until second permanent molar on both sides. An 0.019x0.025-in stainless steel archwire^3^ was designed according to the normal ovoid arch shape of MBT prescription.

The finite element model of mandible for distalizing the dentition consisted of approximately 307,283 three-dimensional tetrahedral elements and 61,618 nodes. Each element was connected to the adjacent elements using the nodes.

### CASE 1 - EXTRA-RADICULAR IMPLANT DISTALIZATION

**Figure 2:**
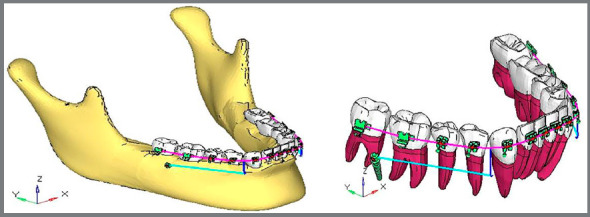
Case 1 - FE model for extra-radicular implant distalization ( ANSYS software ).

A finite model (Fig 2) of bilateral extra-radicular buccal shelf stainless steel mini-implants^18^ (10.0-mm length, 2.0-mm diameter) was placed between the first and second permanent molars. Power hook was attached between the canine and first premolar at a fixed height of 8mm.^19^

### CASE 2 - INTER-RADICULAR IMPLANT DISTALIZATION

**Figure 3:**
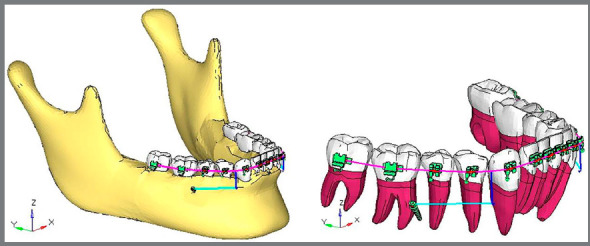
Case 2 - FE model for inter-radicular implant distalization ( ANSYS software ).

A finite element model (Fig 3) of bilateral inter-radicular stainless steel mini-implants^18^ (10.0-mm length; 1.5-mm diameter) was placed between second premolar and first permanent molar. Power hook was attached between canine and first premolar at a fixed height of 8mm.^19^

Each structure was then assigned a specific material property. The different structures in this finite element model included teeth, periodontal ligament, alveolar bone, brackets, archwire and mini-implants. The material properties used in this study were according to values given by Wheelers standard dental anatomy book (Table 1).

**Table 1:** Material properties of different structures involved in the FEA.

Material	Young’s modulus (Mpa)	Poison’s ratio
Cortical bone	13,700	0.30
Cancellous bone	1,370	0.30
Tooth	19,890	0.31
Periodontal ligament (PDL)	50	0.49
Stainless steel	200,000	0.30
Titanium	110,000	0.33
Ni-Ti close coil	35,000	0.33

In this study, all the structures were assumed to be isotropic (for an isotropic material, the properties are same in all directions).

## RESULTS

The result of an analysis is called post-processing. Stresses were calculated and presented in colorful areas, in which different colors represented different stress levels in the deformed state: Red color region of spectrum indicated maximum stresses/displacement, and colors such as orange, yellow, green and blue represented decreasing levels of stresses/displacement, in that order. The results were obtained as distribution of von Mises stresses on the mandibular teeth, mandible, and periodontal ligament. Displacement of the teeth was calculated in three planes, i.e., transverse, sagittal and vertical plane, using X, Y and Z-axes, respectively. The X-axis showed bucco-lingual displacement in transverse plane, Y-axis showed distal displacement in sagittal plane, Z-axis showed displacement in vertical plane.

Finite element models for both the cases were subjected to the distalizing force of 200g (Figs 2 and 3), and the following results were obtained by using the ANSYS v. 12.1 software. 

**Figure 4:**
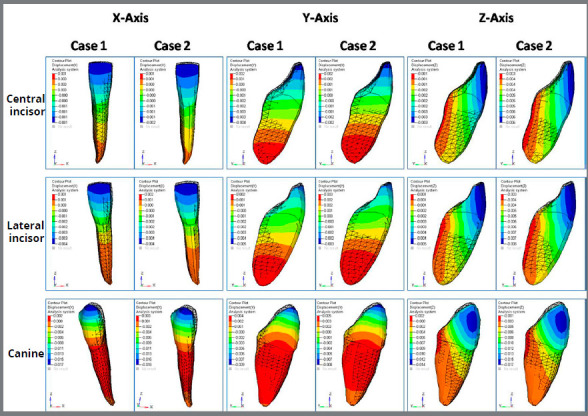
Displacement contour for anterior teeth in X, Y, and Z-axes for extra and inter-radicular mini-implants.

A) Displacement contour for anterior teeth (Fig 4 and Table 2): Displacement contour of anterior teeth showed increased lingual movement in Case 2, in comparison to Case 1, except for the lateral incisor, which showed equal movement (0.004 mm). Case 1 showed increased amount of distalization, in comparison to Case 2. In Z-axis (vertical plane), Case 2 showed more displacement than Case 1. Figure 4 and Table 2 show more controlled movement of anterior teeth in Case 1.

**Table 2:** Displacement contour (in mm) comparison between Case 1 and Case 2.

	X-axis		Y-axis		Z-axis	
	Case 1	Case 2	Case 1	Case 2	Case 1	Case 2
Central incisor	0.001	0.002	0.004	0.002	0.003	0.06
Lateral incisor	0.004	0.004	0.005	0.003	0.005	0.008
Canine	0.017	0.018	0.009	0.008	0.014	0.017
First premolar	0.015	0.017	0.021	0.018	0.015	0.017
Second premolar	0.009	0.005	0.011	0.008	0.004	0.009
First molar	0.002	0.009	0.006	0.005	0.001	0.008
Second molar	0.001	0.007	0.004	0.002	0.001	0.005

**Figure 5:**
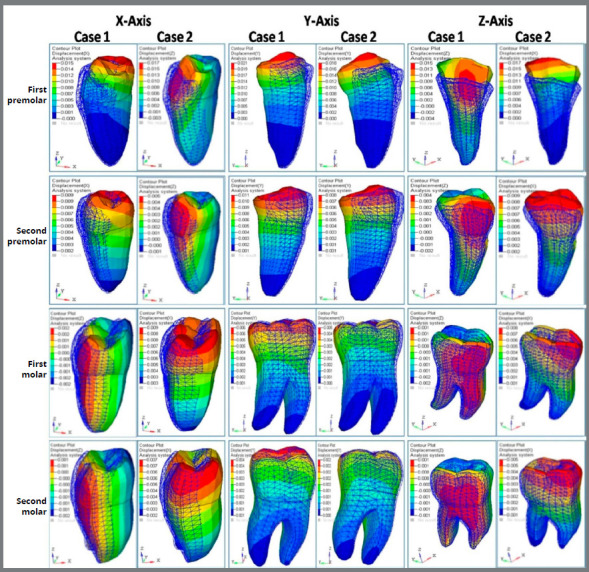
Displacement contour for posterior teeth in X, Y, and Z-axis for extra and inter-radicular mini-implants.

B) Displacement contour for posterior teeth (Fig 5 and Table 2): Displacement contour of posterior teeth showed increased buccal movement in Case 2, in comparison to Case 1, except for the second premolar. Case 1 showed increased amount of distalization, in comparison to Case 2. In Z-axis (vertical plane), Case 2 showed more displacement than Case 1. 

**Figure 6:**
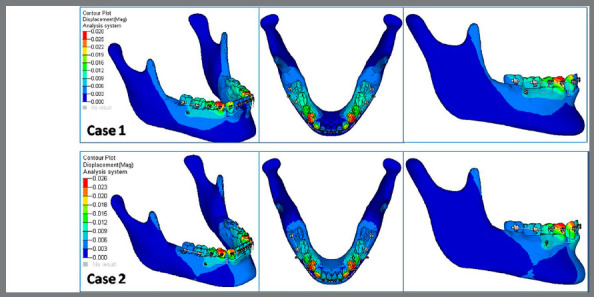
Comparison of displacement contours of mandible in Case 1 and Case 2 ( in mm ).

C) Displacement contour for mandible (Fig 6): Displacement contour of mandible showed increased amount of displacement in Case 1 (0.028 mm), in comparison to Case 2 (0.026 mm). 

**Figure 7:**
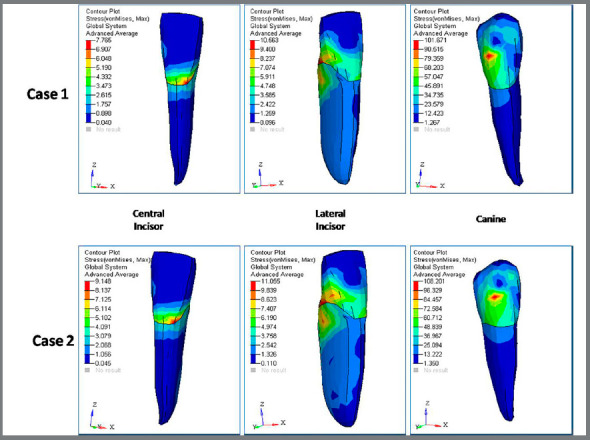
Von mises stress ( in MPa ) contour for anterior teeth, in Case 1 and Case 2.

**Figure 8:**
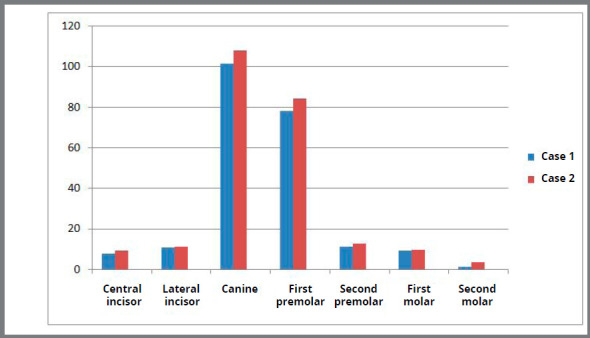
Stress comparison on mandibular teeth.

D) von Mises stress contour for anterior teeth (Figs 7, 8 and Table 3): The stress recorded for central incisors, lateral incisors and canine for Case 1 was 7.76MPa, 10.56MPa and 101.67MPa, respectively. The stress recorded for central incisors, lateral incisors and canine for Case 2 was 9.15MPa, 11.05MPa and 108.20MPa, respectively.

**Figure 9:**
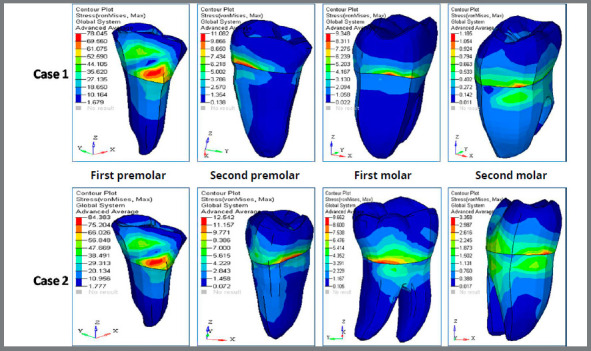
Von mises stress contour (in MPa) for posterior teeth, in Case 1 and Case 2.

E) Von mises stress contour for posterior teeth (Figs 8, 9 and Table 3): The stress recorded at the first premolar for Case 1 and Case 2 was 78.04MPa and 84.38MPa, respectively. The stress distribution at the second premolar for Case 1 and Case 2 was 11.08MPa and 12.54MPa, respectively. The stress observed at the first molar for Case 1 and Case 2 was 9.35MPa and 9.66MPa, respectively. The stress distribution evaluated at the second molar region for Case 1 was 1.18MPa and for Case 2 was 3.36MPa. 

**Table 3:** Von mises stress (in MPa) comparison between Case 1 and Case 2.

Description	Case 1 (MPa)	Case 2 (MPa)
Central incisor	7.76	9.15
Lateral incisor	10.56	11.05
Canine	101.67	108.20
First premolar	78.04	84.38
Second premolar	11.08	12.54
First molar	9.35	9. 66
Second molar	1.18	3.36
Cortical bone	41.93	85.66
Cancellous bone	3.43	3.64
Central incisor at PDL	0.005	0.006
Lateral incisor at PDL	0.016	0.017
Canine at PDL	0.086	0.088
First premolar at PDL	0.081	0.082
Second premolar at PDL	0.009	0.012
First molar at PDL	0.005	0.009
Second molar at PDL	0.001	0.004

**Figure 10:**
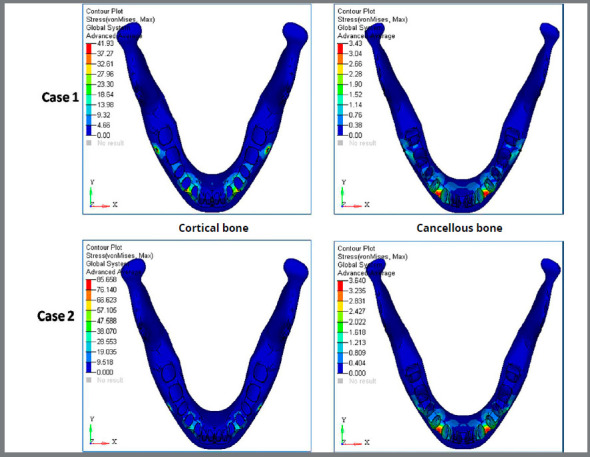
Von mises stress contour (in MPa) for mandible (cortical and cancellous bone) in Case 1 and Case 2.

F) von Mises stress contour for mandible (Fig 10 and Table 3): The stress distribution at the mandible in Case 1 for the cortical bone and cancellous bone was 41.93MPa and 3.43MPa, respectively. The stress distribution at the mandible in Case 1 for the cortical bone and cancellous bone was 85.66MPa and 3.64MPa, respectively. In the Case 2, the amount of stress in mandibular bone was higher than in the Case 1. 

**Figure 11:**
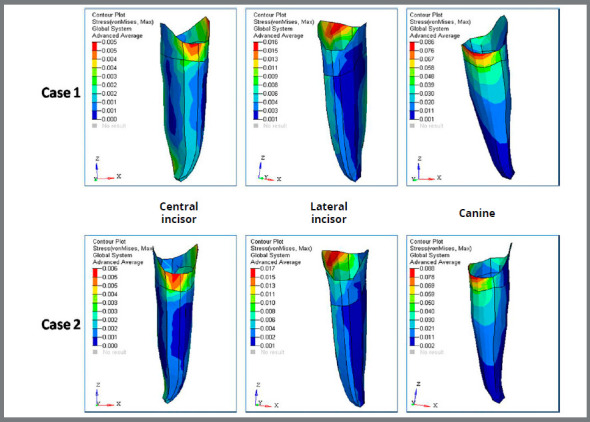
Von mises stress contour (in MPa) for anterior teeth PDL in Case 1 and Case 2.

**Figure 12:**
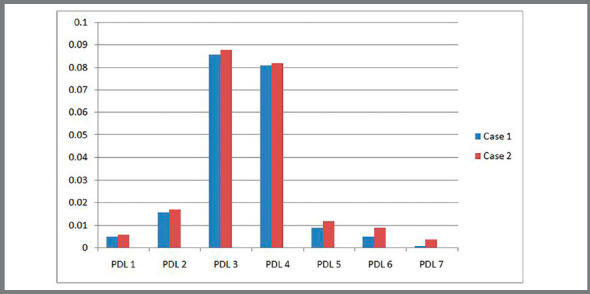
Stress comparison in PDL of mandibular teeth.

G) von Mises stress contour for PDL of anterior teeth (Figs 11, 12 and Table 3): The stress distribution at PDL in the Case 1 for the central incisor, lateral incisor and canine was 0.005MPa, 0.016MPa and 0.086MPa, respectively. The stress distribution at PDL in the Case 2 for the central incisor, lateral incisor and canine was 0.006MPa, 0.017MPa and 0.088MPa, respectively.

**Figure 13:**
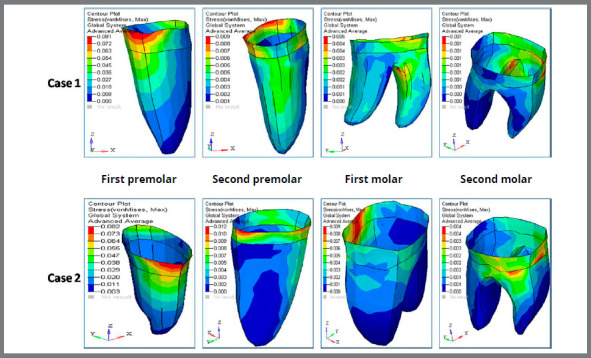
Von mises stress contour (in MPa) for posterior teeth PDL in Case 1 and Case 2.

H) von Mises stress contour for PDL of posterior teeth (Figs 12, 13 and Table 3): The stress evaluated at the PDL for the first premolar for Case 1 was 0.081MPa and for Case 2 was 0.082MPa. The stress at PDL for the second premolar was observed to be 0.009MPa and 0.012MPa for Case 1 and Case 2, respectively. The stress distribution at the PDL for the first molar in Case 1 and Case 2 was observed to be 0.005MPa and 0.009MPa, respectively. The stress distribution evaluated at the PDL for the second molars in Case 1 was 0.001MPa and in Case 2 it was 0.004MPa.

## DISCUSSION

Distalization of the mandibular dentition is an effective treatment for mild to moderate adult Class III malocclusions requiring camouflage therapy. Distalization helps to achieve satisfactory results for the patient, since many patients are reluctant to consent to the surgical option -due to the increased risk and treatment financial cost.^20^ Distalization is the best treatment modality for patients who aim to avoid extraction of the premolars and complicated orthognathic surgery, with long recovery time.^3^ ^,^ ^4^ ^,^ ^7^ ^,^ ^11^ Combining regional acceleratory phenomenon and a mini-implant anchorage system may help in achieving satisfactory results with a shorter treatment time. Extraction of the mandibular third molars immediately before distalization may create a regional acceleratory phenomenon, and help to speed up tooth movements. Puncturing cortical bone in localized areas during mini-implant-assisted retraction may potentially create a regional acceleratory phenomenon. It is observed that mini-implant failure may occur when a regional acceleratory phenomenon is created near the implant site.^1^

Mini-implants can be used in mandibular arch to distalize the mandibular dentition and achieve proper occlusion in Class III patients.^3^ ^,^ ^7^ ^,^ ^11^ The mini-implant placement in retromolar pad area is dependent on the local anatomy of the mandible and the soft tissue thickness in that area. It is often difficult to place retromolar mini-implant successfully for mandibular arch distalization.^21^ Also, it is recommended to place the implant as low as possible near the center of resistance on the mandibular dentition to gain ideal vertical control, which is not possible in case of retromolar pad mini-implant.^22^ Whereas, while placing the buccal shelf implant, increasing the vertical level or insertion angle results in a higher cortical bone thickness and distance from molar root, which is considered a safe and reliable implant placement site.^18^

The current study compared buccal shelf mini-implant distalization and inter-radicular mini-implant distalization, and it was carried out simulating the clinical condition by applying distalizing force to the mandibular dentition. The stress distribution (in MPa) was calculated by using the von Mises criterion.^20^ ^,^ ^21^ Displacements (in mm) for various craniofacial structures were evaluated along the X, Y and Z-axes. The displacement in X-axis indicated the teeth movement in bucco-lingual plane. Both mini-implant types provided force buccally positioned in relation to the center of resistance of the posterior segment, which leads to buccal movement of all teeth involved. In this study, the displacement values were consistent with those found by Chae et al^20^, who explained the distalization biomechanics. The buccal displacement (Fig 5, Table 2) in Case 2 was higher than in Case 1, because the inter-radicular implant was located further away from the center of resistance than the buccal-shelf implant. 

The displacement in Y-axis indicates the movement of the teeth in the mesio-distal direction. Both mini-implant types provided force system that drove the mandibular dentition in distal direction. However, the evaluated displacement (Fig 7, Table 2) of the teeth was higher in the Case 1. In the literature, the maximum distalization achieved with the aid of skeletal anchorage ranged between 2mm and 6mm. The average amount of relapse seen was around 0.3mm. The achieved distalization can sometimes differ at the crown and root level, which can be attributed to the distal tipping that can occur due to various reasons.^20^ ^,^ ^21^ Khan et al^23^ also found that distally placed infrazygomatic crest (IZC) implants show more distalization in the maxillary dentition. In the present study, the distally placed buccal-shelf implant presented higher amount of distalization, in comparison to the mesially placed inter-radicular implant. Hakami et al^24^ described that the distal bodily movement can be attributed to the force applied near the center of resistance of the mandibular arch in combination with a large and stiff working archwire that adequately filled the bracket slot. 

The displacement in Z-axis indicates the intrusion or extrusion of the involved teeth. Both mini-implants provided some amount of intrusion in the posterior segment and extrusion in the anterior segment. However, the vertical displacement (Fig 8, Table 2) of the anterior teeth was higher in Case 2. This evidence suggests greater anti-clockwise rotation of the mandibular arch for Case 2. Chae et al^20^ explained that the center of rotation of the mandibular arch is located near the mesial root of the first molar, at the furcation level. This knowledge allows selection of specific distalization biomechanics based on the skeletal pattern of the patients. Roberts et al^25^ performed a FEA study to describe the distalization biomechanics for a 3-mm molar intrusion: mandibular arch distalization tends to rotate the occlusal plane and close the mandibular angle, thus reducing the vertical anterior facial height, which is beneficial to treat Class III open bite cases.

The von Mises stress values were within the optimum limit of tensile strength of the alveolar bone (135 MPa)^26^ in both cases. The highest amount of stress was 85.65MPa in Case 2, which is far below the ultimate tensile strength, indicating that both cases were clinically safe. Khan et al^23^ and Kushwah et al^27^ also found that during distalization and *en-masse* retraction process, a von Mises stress below 135MPa is safe. 

The stability of the Class III correction also depends on multiple factors such as: tight intercuspation, proper contact alignment in 3D space, optimal functional occlusal plane (perpendicular to the axis of both maxillary and mandibular posterior teeth), resulting in an increase in posterior occlusal support, with minimal and balanced loading of the temporomandibular joint. Proper incisal guidance and freedom of mandibular movements in all direction contribute to treatment stability.^28^

## CLINICAL IMPLICATIONS

From this study, the following recommendations should be considered to obtain better clinical outcomes:

a) This study helps to understand and compare distalization patterns in mandibular arch using extra-radicular and inter-radicular mini-implants.
b) In both cases, generated von Mises stress were below the ultimate tensile stress, indicating safe clinical use.
c) Power hook (8-mm length) helps to apply a line of force near the center of resistance, allowing the translation movement.
d) Case 1 presented greater amount and controlled distalization than Case 2.

## CONCLUSION

In the 3D finite element analysis of stress distribution pattern during distalization of the mandibular dentition using two different types of mini-implant biomechanics in adult patient, the following conclusions can be made:

» The Case 2 (inter-radicular mini-implants) produced higher stress at the tooth, bone and PDL; when compared to the Case 1 (extra-radicular mini-implants), but results were within ultimate tensile limit, being considered clinically safe.
» For anterior teeth, Case 2 showed more movement along Y-axis, except for central incisor, which was the same for both cases.
» Comparing the adverse side-effects in X-axis, a tendency of the posterior segment to move buccally was observed to be more intense in Case 2, except for the second premolar.
» Comparing the adverse side-effects in Z-axis, the tendency of posterior segment intrusion and anterior segment extrusion was observed to be more intense in Case 2.
» In Case 1, there was a significant amount of distance maintained between the roots of the teeth and the mini-implants, avoiding any need to relocate them.

In the vertical dimension, in comparison to the insertion point of the inter-radicular mini-implant (Case 2), the insertion point of the buccal shelf implant (Case 1) was closer to the center of resistance (C_R_) of mandibular dentition; therefore, the line of distalizing force would be closer to the C_R_ in Case 1 and would be nearly parallel to the occlusal plane. Therefore, distalizing the mandibular dentition using a buccal shelf implant will result in less rotation of the occlusal plane, i.e., intrusion of posterior teeth and extrusion of anterior teeth.
